# MTA2 enhances colony formation and tumor growth of gastric cancer cells through IL-11

**DOI:** 10.1186/s12885-015-1366-y

**Published:** 2015-05-02

**Authors:** Chenfei Zhou, Jun Ji, Qu Cai, Min Shi, Xuehua Chen, Yingyan Yu, Zhenggang Zhu, Jun Zhang

**Affiliations:** 1 Department of Oncology, Ruijin Hospital, Shanghai Jiaotong University School of Medicine, No. 197 Ruijin er Road, Shanghai, 200025 China; 2 Department of Surgery, Shanghai Institute of Digestive Surgery, Ruijin Hospital, Shanghai Jiaotong University School of Medicine, No. 197 Ruijin er Road, Shanghai, 200025 China

**Keywords:** Gastric cancer, MTA2, Colony formation, Tumor growth, IL-11

## Abstract

**Background:**

We have preliminarily reported MTA2 expression in gastric cancer and its biological functions by using knockdown cell models, while the molecular mechanisms of MTA2 in regulating malignant behaviors are still unclear.

**Methods:**

MTA2 overexpression models were established by transfection assay in gastric cancer cells BGC-823 and MKN28. Cell proliferation assay, colony formation in soft agar, wound-healing assay and transwell migration assay were performed with MTA2 overexpression and negative control (NC) cells. Subcutaneous xenografts and pulmonary metastasis models by BGC-823/MTA2 and BGC-823/NC cells were used to observe the capacity of growth and metastasis *in vivo*. Differential gene expression in MTA2 knockdown and overexpression cells was analyzed by microarrays. IL-11, which demonstrated as differential expression in microarray, was detected by real-time PCR, western blot, ELISA and immunohistochemistry staining. Recombinant human IL-11 (rhIL-11) was administrated in cell proliferation and colony formation as rescue assay.

**Results:**

The numbers of colonies in soft agar were significantly more in BGC-823/MTA2 and MKN28/MTA2 cells, comparing with those in their NC cells. Capabilities of cell proliferation, wound-healing and cell migration were not significantly changed in MTA2 overexpression cells. The sizes of subcutaneous xenografts and pulmonary metastases of BGC-832/MTA2 cells were significantly larger than those in BGC-823/NC group. Differential expression of IL-11 was identified by genome expression microarray both in MTA2 knockdown and overexpression cells. IL-11 expression was elevated in BGC-823/MTA2 cells, whereas reduced in SGC-7901/shMTA2 cells. Administration of rhIL-11 recovered colony formation capacity of SGC-7901/shMTA2 cells.

**Conclusions:**

MTA2 overexpression enhances colony formation and tumor growth of gastric cancer cells, but not plays important role in cancer cell migration and metastasis. IL-11 is one of the downstream effectors of MTA2 in regulating gastric cancer cells growth.

**Electronic supplementary material:**

The online version of this article (doi:10.1186/s12885-015-1366-y) contains supplementary material, which is available to authorized users.

## Background

Gastric cancer is one of the most malignant gastrointestinal neoplasms in the world [1]. In the past decades, although its incidence is decreasing worldwide, gastric cancer is still one of the leading causes of cancer-specific mortality in mainland of China [2]. To improve the outcome of gastric cancer patients, mechanisms of tumorigenesis and progression are under investigation, and intent to clinical translation. However, due to the multiple molecular aberrances and high heterogeneity, relevant molecular events of gastric cancer cells are far from elucidated [3].

Metastasis associated 1 family, member 2 (MTA2) gene is classified to metastasis associated gene family [4]. Like MTA1, aberrant expression of MTA2 has been reported in several tumors, including ovarian, lung and pancreatic cancer [5-7]. We have previously reported that MTA2 gene was overexpressed in gastric cancer tissues, correlating with tumor invasion, lymph node metastasis, and advanced TNM stage. MTA2 knockdown significantly inhibited gastric cancer cell invasion and metastasis [8]. Yet, its molecular mechanisms are still unclear.

As a component of the nucleosome remodeling and histone deacetylation (NuRD) complex, MTA2 is necessary for assembly of a catalytically active histone deacetylase (HDAC) complex. HDAC activity is significantly impaired without MTA2 [9]. Nucleosome remodeling and histone deacetylation are the major processes for gene transcriptional regulation [10]. Furthermore, MTA2 has been reported to form transcription complexes with some transcription factors, such as ERα and Twist [11]. Those results indicate that impacts of MTA2 on biological function might via regulating specific genes which were related with tumor malignant behaviors.

In present study, to validated MTA2 biological function, MTA2 overexpression cell models established by MTA2 low expression gastric cancer cells were investigated. Differential gene expression in MTA2 overexpression and knockdown cells was analyzed to identify its downstream effectors. Meanwhile, the biological functions of MTA2-downstream effectors were reversely confirmed through rescue assay.

## Methods

### Cell lines and reagents

Gastric cancer cell lines BGC-823 and MKN28 were preserved in Shanghai Institution of Digestive Surgery. MTA2 knockdown cell SGC-7901/shMTA2 and its negative control cell SGC-7901/NC were described in previous study [8]. All cells were cultured at 37°C with 5% CO_2_ in RPMI-1640 medium with 10% fetal calf serum. Selection antibiotics, puromycin or hygromycin, were added into medium if necessary.

Puromycin (Sigma) and hygromycin (Sigma) were respectively dissolved by phosphate buffered saline (PBS) at concentrations of 1 mg/ml and 200 mg/ml. Recombinant human interleukin-11 (rhIL-11, R&D systems) was reconstituted following user manual at a concentration of 50 μg/ml. SAHA (Selleckchem) was dissolved by DMSO at a concentration of 200 mM. All reagents were divided into aliquots, and were stored at -80°C.

### Establishment of MTA2 overexpression cell models

Full-length cDNA sequence of MTA2 gene (Gene ID: 9219) was cloned and constructed into vector with Flag-tag. MTA2 plasmid and its negative control were packaged into lentivirus, and were used to infect BGC-823 and MKN28 cells, respectively. Stable transfected cells were selected by medium with hygromycin (200 μg/ml). MTA2 overexpression cell models were named as BGC-823/MTA2 and MKN28/MTA2, and negative controls were named as BGC-823/NC and MKN28/NC.

### RNA extraction and real-time PCR

Total cell RNA was extracted by Trizol reagent, and their concentrations were determined by microplate spectrophotometer (BioTek). Reverse transcription was performed following protocol of Applied Biosystems. Real-time PCR was performed by ABI Prism 7900HT sequence detection system (Applied Biosystems). Primers for real-time PCR were MTA2: F-TGT ACC GGG TGG GAG ATT AC, R-TGA GGC TAC TAG AAA TGT CCC TG; IL-11: F- ACA GCT GAG GGA CAA ATT CC, R- AGC ACA CCT GGG AGC TGT AG. GAPDH was used as reference gene.

### Western blot

Whole cell proteins were extracted by RIPA reagent (Solarbio), and protein concentrations were determined by DC protein assay (Bio-Rad). Denatured samples were fractionated by SDS-PAGE gel and transferred to PVDF membranes. Membranes were blocked by skim milk for 1 h at room temperature. Primary antibodies, MTA2 (1:1000, BETHYL), IL-11 (1:200, Santa cruz), β-actin (1:5000, Sigma) and GAPDH (1:5000, KANGCHEN), were incubated overnight at 4°C. Fluorescent secondary antibodies (1:15000, LI-COR) were incubated at room temperature for 1 h. Protein bands were visualized by infrared imaging system (LI-COR).

### Immunofluorescence

Attached cells were fixed by 4% paraformaldehyde for 30 min, and penetrated by Triton X-100 for 15 min, then blocked by BSA for 30 min. DYKDDDK Tag (9A3) mouse mAb (1: 100, Cell signaling) was incubated at room temperature for 1 h. After rinsed by PBS, Anti-mouse IgG1 FITC (1:100, eBioscience) was incubated for 30 min. Slides were mounted by VECTASHIELD Mounting Medium with DAPI (Vector Labs). Images were captured by confocal microscope, with Zeiss LSM Image Examiner (Carl Zeiss).

### Cell proliferation assay

One thousand cells per well were plated into 96-well plates, and were cultured overnight. CCK-8 (Dojindo) were added and incubated at 37°C for 2 h. Optical density value was detected by microplate spectrophotometer (BioTek). Cell growth curves were drawn.

### Soft agar colony formation assay

Two milliliter medium with 0.4% agar was used as bottom gel. One thousand cells were suspended in 4 ml medium with 0.2% agar, and were poured on bottom gel. After 2 weeks, colonies were stained by MTT. Number of colonies was counted at low power lens of microscopy. Size of colonies was analyzed by Colony Counter software (Tanon). RhIL-11 was added at a concentration of 100 ng/ml.

### Wound healing assay and migration assay

Cells were cultured as monolayer in 12-well plates. On reaching more than 95% confluent, wounds were scratched by pipette tips. After washed by PBS, wounds were photographed every 24 h. Cells were cultured by serum-free medium during the process.

Fifty thousand cells suspended in 100 μl serum-free medium were added into upper chambers of Transwell (8 μm for 24-well plate, Millipore), and full medium were added into lower chambers. After 24 h, cells were fixed by formalin, and stained by 0.1% crystal violet.

### Nude mouse xenografts

BGC-823/MTA2 and BGC-823/NC cells were studied *in vivo*. All mouse experiments were approved by the Animal Care and Use Committee and conducted an accord with the Guide for the Care and Use Laboratory Animals of Ruijin Hospital, Shanghai Jiaotong University School of Medicine. Eight 4-week-old male Balb/c nude mice (Institute of Zoology, Chinese Academy of Sciences) were randomly divided into two groups before establishment of subcutaneous xenografts. One million cells in 100 μl PBS were injected into subcutaneous tissues of right side back. After tumor length exceeded 4 mm, tumors were measured every 7 days for 4 weeks, and tumor volume was calculated by following formula: V = (Width^2^ × Length)/2. At 5^th^ week, tumor nodules were collected, and fixed by 10% formalin.

In experimental metastasis assay, twenty Balb/c nude mice (Institute of Zoology, Chinese Academy of Sciences) were randomly divided into two groups. One million BGC-823/MTA2 or BGC-823/NC cells in 100 μl PBS were injected through tail vein, respectively. One mouse of each group was sacrificed after one month to assess metastasis formation. After two months, all mice were dissected. Pulmones were fixed by 10% formalin. Metastatic lesions were counted in gross specimens, and were confirmed by HE staining.

### Immunohistochemistry staining (IHC)

Fixed tumor nodules were embedded by paraffin, and were made into 4 μm-thick slices. IHC were performed following EnVision two-step procedure of Dako™ REAL™ Envision Detection System (Dako). Primary antibodies included MTA2 (1:200, Santa Cruz), Ki-67 (1:50, Dako), IL-11 (1:100, Santa Cruz). Slices were visualized by diaminobenzidine.

### Microarray data

Differential gene expression between BGC-823/NC and BGC-823/MTA2, SGC-7901/NC and SGC-7901/shMTA2 cells was analyzed, respectively. Total RNA of cells were extracted by Trizol reagent. Whole human genome microarray 4 × 44 K (Design ID: 014850, Agilent technologies) was used to detect gene expression profile following manufacturer’s instructions. Raw data were normalized by Quantile algorithm using Gene Spring Software 11.0 (Agilent technologies). Genes with fold change >2 or <0.05 and p-value <0.05 were identified. Microarray data are available in the ArrayExpress database (www.ebi.ac.uk/arrayexpress) under accession number E-MTAB-3469.

### Enzyme-linked immunosorbent assay (ELISA)

Supernatants of SGC-7901/NC and SGC-7901/shMTA2 cells, BGC-823/NC and BGC-823/MTA2 cells were collected, and were centrifuged to remove cell debris. Total levels of IL-11 protein were measured by using human IL-11 antibody (R&D system, MAB618). IL-11 levels were normalized to amount of mRNA in each sample.

### Statistics

Independent-samples *t* test, Mann-Whitney U test and one-way ANOVA test (LSD post-hoc) were used to analyze quantitive data. *P*-value <0.05 was considered as statistical significant (*: P < 0.05; **: P < 0.01). The error bars in all figures represented standard deviation. All tests were preformed by SPSS 13.0 software (SPSS Inc.).

## Results

### MTA2 overexpression promoted cell colony formation in soft agar

**Figure 1:**
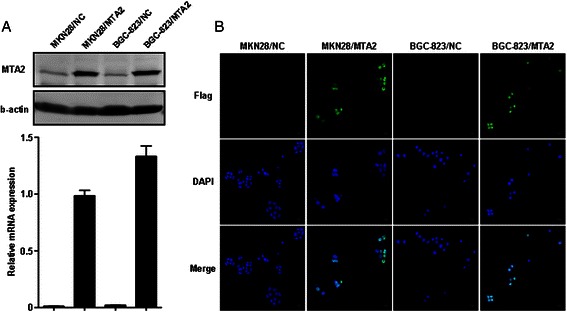
Establishment of MTA2 overexpression gastric cancer cell models. **A**: MTA2 expression in BGC-823 and MKN28 cells was detected by real-time PCR and western blot. **B**: Exogenous MTA2 protein was localized in nucleus (immunofluorescence). “Immunofluorescence staining demonstrated that exogenous MTA2 protein was localized in cell nucleus (Figure 1B).” “High magnification image was provided in Additional file 1: Figure S1.”

Introduction of MTA2 into BGC-823 and MKN28 cells was confirmed by both real-time PCR and western blot (Figure 1A). Immunofluorescence staining demonstrated that exogenous MTA2 protein was localized in cell nucleus (Figure 1B).

**Figure 2:**
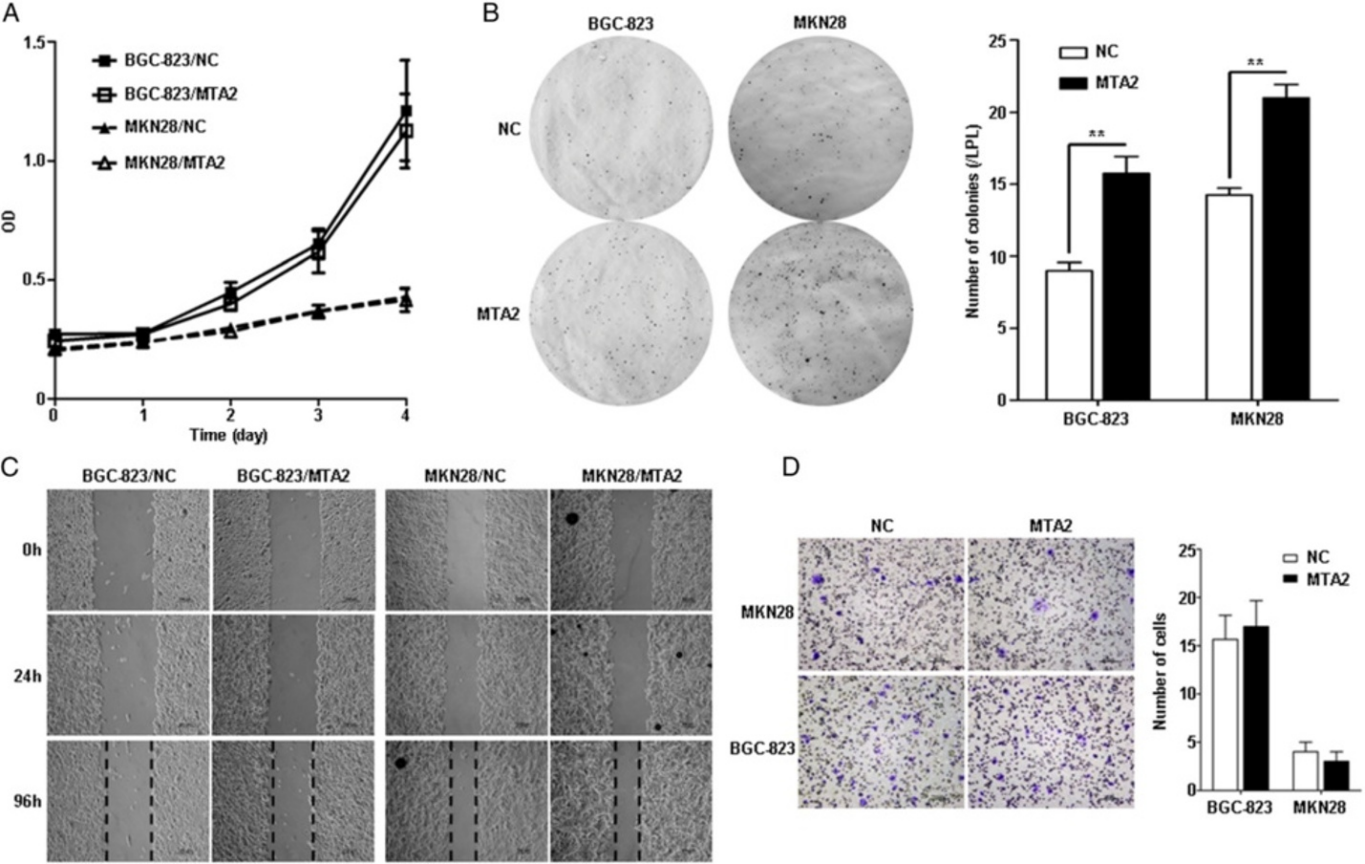
MTA2 overexpression enhanced cell colony formation in soft agar. **A**: Growth curves were similar between MTA2 overexpression and NC cells. **B**: The number of colonies formed in soft agar was more in MTA2 overexpression cells than it in NC cells. **C**: Wound-healing courses were similar between MTA2 overexpression and NC cells. **D**: The numbers of cells passed though membrane were not different between MTA2 overexpression and NC cells.

Growth curves of BGC-823/MTA2 and MKN28/MTA2 cells were similar, as compared with those in NC cells (Figure 2A). In soft agar colony formation assay, more colonies were observed in MTA2 overexpression cells (MTA2 vs. NC, BGC823: 15.8 ± 2.4 vs. 9.0 ± 1.2, *P* = 0.002; MKN28: 21.0 ± 1.8 vs. 14.3 ± 1.0, *P* = 0.001, Figure 2B).

No differences were found between BGC-823/MTA2 and BGC-823/NC cells in wound-healing courses, as well as those in MKN28/MTA2 and its NC cells (Figure 2C). In migration assay, the numbers of cells passed through membrane were also similar between MTA2 overexpression and NC cells (MTA2 vs. NC, BGC-823: 17.0 ± 2.6 vs. 15.7 ± 2.5, *P* > 0.05; MKN28: 3.0 ± 1.0 vs. 4.0 ± 1.0, *P* > 0.05, Figure 2D).

### MTA2 overexpression enhanced xenografts and pulmonary metastases growth

**Figure 3:**
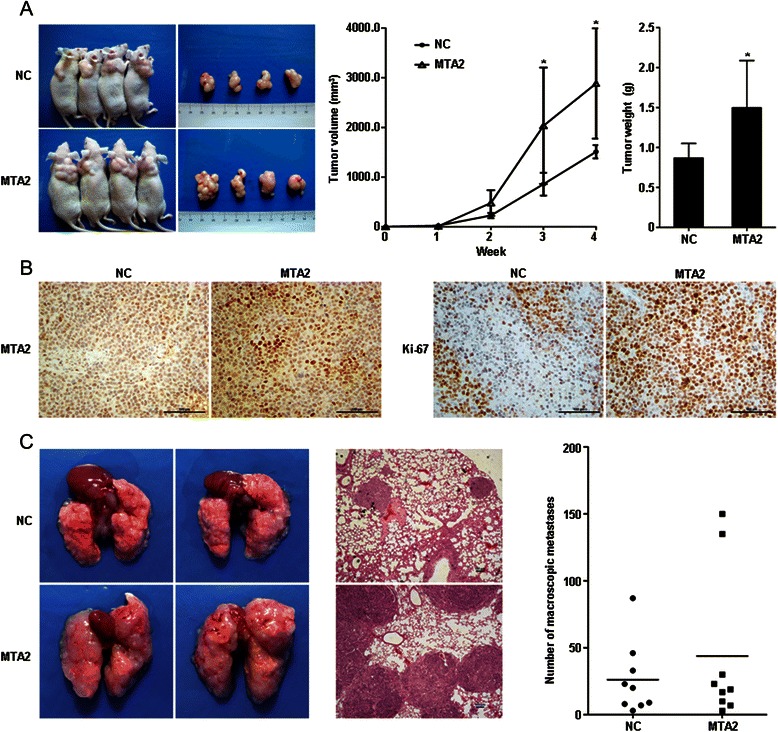
MTA2 overexpression in BGC-823 cell enhanced growth of subcutaneous xenografts and pulmonary metastases. **A**: Size and weight of subcutaneous xenografts in BGC-823/MTA2 group were larger than those in BGC-823/NC group. **B**: Ki-67 staining in BGC-823/MTA2 group was stronger than it in BGC-823/NC group. **C**: Size of pulmonary metastases in BGC-823/MTA2 group was larger than those in BGC-823/NC group, while numbers of lesions were not significantly different.

Growth rate of subcutaneous xenografts in BGC-823/MTA2 group was significantly faster than that in BGC-823/NC group. At fourth week, tumor volume and weight were significantly larger in BGC-823/MTA2 group than those in NC group (volume: 2885.7 ± 1109.8 mm^3^ vs. 1509.6 ± 133.2 mm^3^, *P* = 0.021; weight: 1.51 ± 0.58 g vs. 0.86 ± 0.17, *P* = 0.038, Figure 3A). Ki-67 staining was stronger in BGC-823/MTA2 xenografts than that in NC (Figure 3B).

Pulmonary metastases were observed in all mice. In BGC-823/MTA2 group, the volume of metastatic lesions was larger than those in BGC-823/NC group (Figure 3C). However, the number of metastases was no difference between those two groups (MTA2 vs. NC: 43.8 ± 56.7 vs. 26.2 ± 26.7, *P* > 0.05, Figure 3C).

### IL-11 was identified as a differential expressed gene by microarray

Differential gene expression between BGC-823/MTA2 and BGC-823/NC cells as well as SGC-7901/shMTA2 and SGC-7901/NC cells were analyzed. Overlapped differential genes between those two data sets were identified. IL-11 demonstrated the most significant fold changes in both data sets (Table 1).

**Table 1:** **Differential gene expression in MTA2 overexpression and knockdown cells**

Gene	MTA2 overexpression		MTA2 knockdown		Description
	Fc	P value	Fc	P value	
IL-11	7.3145	7.74E-06	0.2631	0.0054	Interleukin 11
TXNIP	2.8848	0.0006	0.0176	1.54E-05	Thioredoxin interacting protein
AIM1L	0.4801	0.0071	2.1508	0.0013	Absent in melanoma 1-like
KLK1	0.4736	0.0039	2.7208	0.0009	Kallikrein 1
RAET1E	0.4116	0.0027	6.1737	0.0017	Retinoic acid early transcript 1E
KCNQ2	0.3869	0.0009	2.6920	0.0001	Potassium voltage-gated channel, KQT-like subfamily, member 2
NES	0.3735	0.0015	2.9488	0.0001	Nestin
HSPA2	0.3183	0.0009	2.1676	0.0068	Heat shock 70 kDa protein 2
SUSD2	0.3074	0.0017	2.5593	3.79E-06	Sushi domain containing 2
MFAP5	0.3055	0.0049	3.1193	0.0043	Microfibrillar associated protein 5
OLAH	0.2559	8.55E-05	2.3232	0.0032	Oleoyl-ACP hydrolase
ALPPL2	0.2195	0.0004	2.0811	0.0004	Alkaline phosphatase, placental-like 2

**Figure 4:**
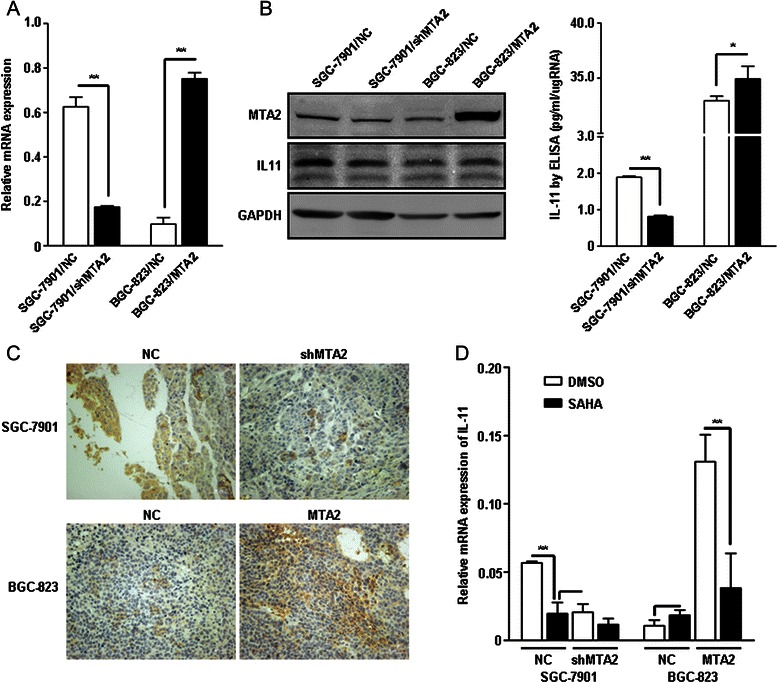
IL-11 expression related with MTA2 in SGC-7901 and BGC-823 cells. **A**: IL-11 expression was detected by real-time PCR. **B**: IL-11 protein was detected by western blot and ELISA. **C**: IL-11 expression in SGC-7901 and BGC-823 xenografts was detected by IHC. **D**: IL-11 mRNA expression was reduced by SAHA in both SGC-7901/NC cell and BGC-823/MTA2 cell.

IL-11 mRNA was significantly changed in SGC-7901/shMTA2 and BGC-823/MTA2 cells, as the results of microarray (P < 0.01, Figure 4A). IL-11 protein was also reduced in SGC-7901/shMTA2 cells, and slightly elevated in BGC-823/MTA2 cells by western blot, as well as in ELISA test (SGC-7901/NC vs. SGC-7901/shMTA2: 1.89 ± 0.33 vs. 0.81 ± 0.35, P < 0.01; BGC-823/NC vs. BGC-823/MTA2: 32.97 ± 0.40 vs. 34.93 ± 1.15, P = 0.049, Figure 4B). IHC showed that IL-11 expression was reduced in SGC-7901/shMTA2 xenografts, and was significantly increased in BGC-823/MTA2 xenografts (Figure 4C).

### IL-11 expression was reduced by HDAC inhibitor SAHA

After SAHA treatment (4 μM for 24 h), IL-11 mRNA in SGC-7901/NC cells was significantly decreased (*P* < 0.01), and similar with the level in SGC-7901/shMTA2 cells treated by DMSO. In BGC-823/NC cells, IL-11 mRNA was not changed by SAHA (*P* > 0.05), while its expression in BGC-823/MTA2/SAHA group was significantly decreased (*P* < 0.01, Figure 4D).

### IL-11 recovered colony formation capacity of MTA2 knockdown cells

**Figure 5:**
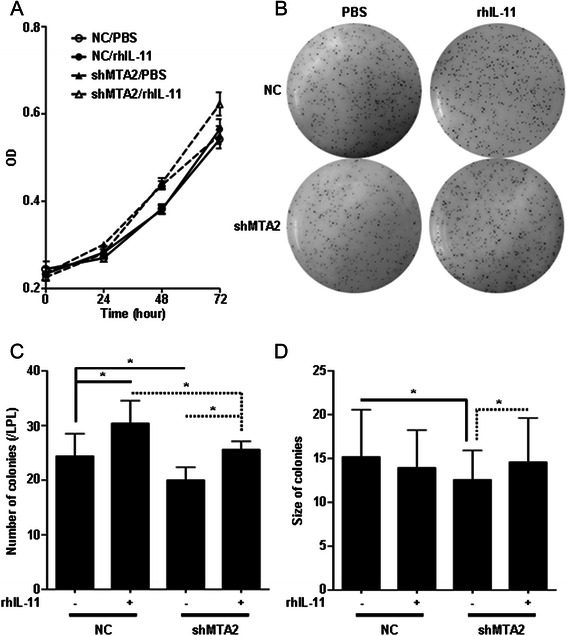
IL-11 recovered colony formation capacity of SGC-7901/shMTA2 cell. **A**: Growth curves of SGC-7901/NC and SGC-7901/shMTA2 cells were not affected by rhIL-11 treatment. **B**: Colony formation in soft agar was assessed after rhIL-11 treatment. **C**: Number of colonies in SGC-7901/shMTA2/IL-11 group was more than it in SGC-7901/shMTA2/PBS group, and similar with SGC-7901/NC/PBS group. **D**: Size of colonies in SGC-7901/shMTA2/IL-11 group was larger than it in SGC-7901/shMTA2/PBS group.

SGC-7901/shMTA2 and SGC-7901/NC cells were treated by rhIL-11 (100 ng/ml). In cell proliferation assay, cell growth rates were not significantly changed among cells treated by rhIL-11 or PBS (Figure 5A).

In colony formation assay, the colony number of SGC-7901/shMTA2 cells treated by rhIL-11 was more than those treated by PBS (SGC-7901/shMTA2: rhIL-11 vs. PBS, 25.6 ± 1.5 vs. 20.0 ± 2.3, *P* < 0.01), and was similar with the number of SGC-7901/NC/PBS group (24.4 ± 4.1, *P* = 0.566). The number of colonies of SGC-7901/NC cells treated by rhIL-11 was also more than those treated by PBS (SGC-7901/NC: rhIL-11 vs. PBS, 30.4 ± 4.2 vs. 24.4 ± 4.1, *P* < 0.01, Figure 5C). The average size of colonies was also larger in SGC-7901/shMTA2/rhIL-11 group than that in SGC-7901/shMTA2/PBS group (14.6 ± 5.0 vs. 12.6 ± 3.3, *P* = 0.004, Figure 5D).

## Discussion

Herein, we found that MTA2 overexpression in gastric cancer increased colony formation *in vitro* and tumor growth *in vivo*, including either subcutaneous xenografts or pulmonary metastases. By genome expression analysis, IL-11 expression was found to be in accordance with down- and up-regulation of MTA2. Recombinant human IL-11 treatment could recover colony formation capacity of MTA2 knockdown cells.

The biological functions of MTA2 in cancer have not been fully investigated. MTA2 was first identified in quickly dividing cells in cervical cancer [9]. In non-small cell lung cancer, ki-67 index in MTA2 positive tissues was higher than that in negative ones [6]. MTA2 overexpression could increase colony formation of osteosarcoma cells SaoS-2 by inhibiting p53 activity, and also enhanced ER-α positive breast cancer cells growth [12,13]. On the other hand, in mouse breast cancer 4 T1 cells and glioma cells, it was reported that MTA2 knockdown impaired cell invasion and metastasis [11,14]. Those results showed that MTA2 played role in various malignant behaviors, including proliferation, invasion and metastasis. However, few studies assessed the biological function of MTA2 in both overexpression and knockdown manners.

We have previously reported that MTA2 knockdown in SGC-7901 and AGS gastric cancer cell lines with high MTA2 expression inhibited gastric cancer cell invasion and metastasis. Although attenuated cell colony formation and subcutaneous tumor growth were observed, these phenomena were attributed to the impaired cell invasion. Changes of cells morphology and reduced CD24 and MYLK expression further supported the conclusion that depression of cell mobility could be the major consequence of MTA2 knockdown in gastric cancer cells [8].

To verify those phenomena, cell models based on gastric cancer cells with different genetic background were preferred to be used. Therefore, BGC-823 and MKN28 cell lines with low MTA2 expression was used to establish MTA2 knock-in cell models. From the results, we found that enhanced cell migration was not observed in MTA2 overexpression cells. Although CD24 and MYLK were identified by genome expression analysis in MTA2 knockdown cells as pervious reported, those two genes was not found in our MTA2 overexpression date set. Instead of cell invasion, colony formation in soft agar and tumor growth *in vivo* were significantly enhanced by MTA2 overexpression, contrast to MTA2 knockdown. Positive correlation between Ki-67 and MTA2 was detected in both MTA2 knockdown and overexpression xenografts. Those results demonstrated that in gastric cancer cells, MTA2 was strongly related with cell colony formation and tumor growth. MTA2 participated in gastric cancer cell invasion, but might not be a dominant regulator.

The different results between cell proliferation assay and *in vivo* experiments indicated that interactions between tumor cells and its microenvironment could correlate with growth promoting effect of MTA2 [15]. Intercellular contact, growth factors, cytokines and extracellular matrix, all play important roles in tumor growth of gastric cancer [16]. In present study, IL-11 expression was found related with MTA2 in gastric cancer cells by genome expression analysis, and was validated in both cell models and xenografts tissues.

IL-11 belongs to IL-6 cytokine family, which consisted of IL-6, IL-11, IL-27, IL-30, IL-31, oncostatin M, and others, and it can be secreted by cancer-associated fibroblasts, myeloid cells, and tumor cell itself [17,18]. Aberrant expression of IL-11 and its receptor IL-11Rα was found in gastric cancer tissues, and correlated with Lauren’s classification, tumor invasion and vessel infiltration [19]. In transgenic mice model carrying gp130^Y757F/Y757F^, the IL-11 receptor with substitution of tyrosine (Y) 757 by phenylalanine (F), spontaneous gastric tumorigenesis is found. This mutation abolished negative feedback of gp130, resulting consistent activation of IL-11 downstream signaling pathway [20]. Gastric tumor formed in gp130^Y757F/Y757F^ mice could be significantly abrogated by IL-11Rα knock-out and also by IL-11 signaling antagonist. Those results demonstrated that IL-11 was one of the dominant factors in gastric cancer development and progression [21,22].

To validate whether IL-11 was involved in cell colony formation regulated by MTA2, rhIL-11 was used to treat MTA2 knockdown cells in present study. Administration of rhIL-11 recovered colony formation ability of MTA2 knockdown cells and could further enhance it in NC cells, while cell proliferation was not effected by rhIL-11. Colony formation of BGC-823 cell could also enhanced by IL-11 treatment (Additional file 2: Figure S2). On the other hand, by using antibody to neutralize IL-11 function in BGC-823/MTA2 cell, its colony formation was impaired (Additional file 3: Figure S3). The rescue assay suggested that MTA2 promoting gastric cancer cell colony formation might partially through IL-11 as a downstream effector.

The mechanisms of MTA2 in regulating gene expression are currently obscure. Because of its role in NuRD complex to maintain HDAC activity, the impact of MTA2 on IL-11 expression could partially via HDAC pathway. Therefore, we used HDAC inhibitor to simulate the status of MTA2 knockdown. After SAHA treatment, IL-11 expression was significantly reduced in SGC-7901/NC cell, and its level was similar to MTA2 knockdown cells. IL-11 expression in MTA2 overexpression cells was also decreased. Those results indicated that HDAC activity regulated by MTA2 might involve in regulating IL-11 expression.

Not only participates in NuRD complex formation, MTA2 could also form complexes with some transcription factors, resulting in gene transcriptional repression. Interaction of MTA2 and Twist participated in repressing E-cadherin promoter activity [11]. Binding with ERα, MTA2 could repress its transcriptional activity in breast cancer cells [13]. In present study, increased expression of IL-11 in MTA2 overexpression cells indicated that MTA2 could promote specific gene expression, and its mechanism should be investigated in further studies.

Besides IL-11, several genes were identified by genome expression analysis. Some of those genes had been investigated in cancer cells, while the functions of others were still unclear. TXNIP is an endogenous antagonist of TRX, and can regulate cellular redox equilibrium by repressing TRX activity [23]. Expression of RAETI1E was correlated with poor prognosis of ovarian cancer patients [24]. Biological functions of HSPA2 had also been investigated in several tumors [25,26]. The roles of those genes in malignant behaviors regulated by MTA2 should be further explored.

## Conclusions

MTA2 overexpression enhances colony formation and tumor growth of gastric cancer cells, but does not promote tumor migration and metastasis. IL-11 is one of the downstream effectors of MTA2 in regulating gastric cancer cells growth.

## Additional files

Additional file 1: Figure S1. Immunofluorescence staining of MTA2 in MTA2 overexpression cells. Immunofluorescence staining showed that exogenous MTA2 localized in cellular nucleus.
Additional file 2: Figure S2. Colony formation of BGC-823 cells was enhanced by IL-11. BGC-823 cells in soft agar were treated by IL-11 and PBS, respectively. The method was described in manuscript. Results showed that number and size of colonies in BGC-823/IL-11 group was more than those in BGC-823/PBS group (IL-11 vs. PBS, number: 12.8 ± 2.4 vs. 9.2 ± 1.3, *P* = 0.018; size 70.0 ± 47.8 vs. 50.0 ± 34.8, *P* < 0.001).
Additional file 3: Figure S3. Colony formation of BGC-823/MTA2 cells was reduced by IL-11 antibody. BGC-823/MTA2 cells in soft agar were treated by IL-11 antibody (R&D systems, #22626, 10 μg/ml) and PBS, respectively. Results showed that number of colonies in BGC-823/MTA2/anti-IL11 group was less than those in BGC-823/MTA2/PBS group (5.8 ± 0.8 vs. 13.6 ± 3.4, *P* = 0.001). Size of colonies between those two groups was similar (30.4 ± 19.4 vs. 36.2 ± 28.9, *P* = 0.194).

## Abbreviations

NC: Negative control
MTA2: Metastasis associated 1 family, member 2
NuRD: Nucleosome remodeling and histone deacetylation
HDAC: Histone deacetylase
