# Supplementary figures and images for: MTA2 enhances colony formation and tumor growth of gastric cancer cells through IL-11
Source: BMC Cancer. 2015 May 2;15:343. doi: 10.1186/s12885-015-1366-y (PMC4419442; doi:10.1186/s12885-015-1366-y)

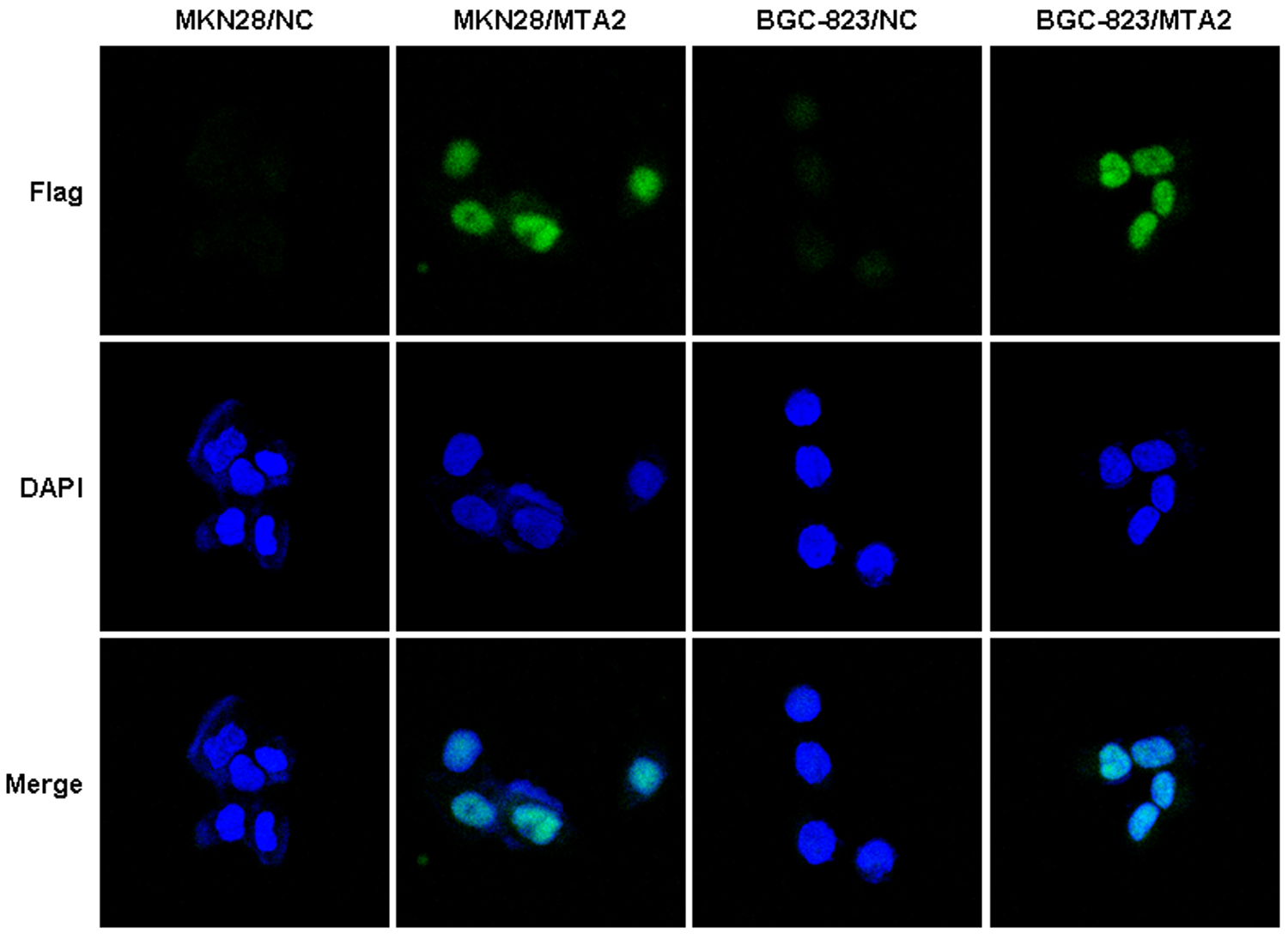

Supplement: Additional file 1: Figure S1. — Immunofluorescence staining of MTA2 in MTA2 overexpression cells. Immunofluorescence staining showed that exogenous MTA2 localized in cellular nucleus. [file 12885_2015_1366_MOESM1_ESM.tiff]

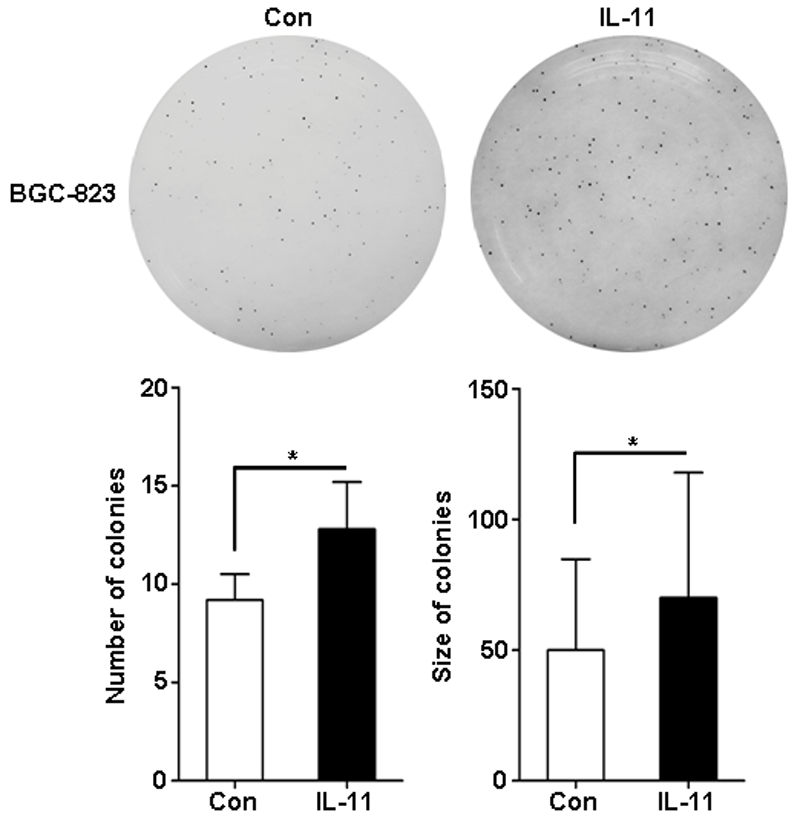

Supplement: Additional file 2: Figure S2. — Colony formation of BGC-823 cells was enhanced by IL-11. BGC-823 cells in soft agar were treated by IL-11 and PBS, respectively. The method was described in manuscript. Results showed that number and size of colonies in BGC-823/IL-11 group was more than those in BGC-823/PBS group (IL-11 vs. PBS, number: 12.8 ± 2.4 vs. 9.2 ± 1.3, P = 0.018; size 70.0 ± 47.8 vs. 50.0 ± 34.8, P < 0.001). [file 12885_2015_1366_MOESM2_ESM.tiff]

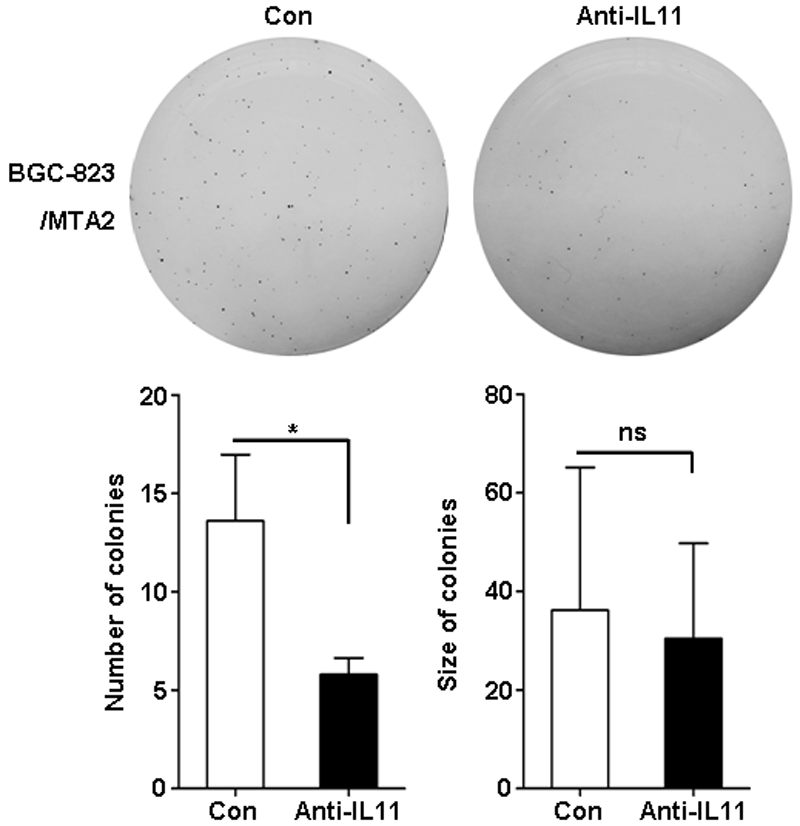

Supplement: Additional file 3: Figure S3. — Colony formation of BGC-823/MTA2 cells was reduced by IL-11 antibody. BGC-823/MTA2 cells in soft agar were treated by IL-11 antibody (R&D systems, #22626, 10 μg/ml) and PBS, respectively. Results showed that number of colonies in BGC-823/MTA2/anti-IL11 group was less than those in BGC-823/MTA2/PBS group (5.8 ± 0.8 vs. 13.6 ± 3.4, P = 0.001). Size of colonies between those two groups was similar (30.4 ± 19.4 vs. 36.2 ± 28.9, P = 0.194). [file 12885_2015_1366_MOESM3_ESM.tiff]
